# Role of partial molar enthalpy of oxides on Soret effect in high-temperature CaO–SiO_2_ melts

**DOI:** 10.1038/s41598-018-33882-1

**Published:** 2018-10-19

**Authors:** Masahiro Shimizu, Jun Matsuoka, Hiroshi Kato, Takeyuki Kato, Masayuki Nishi, Heidy Visbal, Kohji Nagashima, Masaaki Sakakura, Yasuhiko Shimotsuma, Hiroki Itasaka, Kazuyuki Hirao, Kiyotaka Miura

**Affiliations:** 1 Graduate School of Engineering, Kyoto University, Kyoto, Japan; 2 School of Engineering, University of Shiga Prefecture, Hikone, Japan; 3 Optoelectronics Research Centre, University of Southampton, Southampton, UK; 4 National Institute of Advanced Industrial Science and Technology, Nagoya, Japan; 5 Center for the Promotion of Interdisciplinary Education and Research, Kyoto University, Kyoto, Japan

## Abstract

The Soret effect or thermodiffusion is the temperature-gradient driven diffusion in a multicomponent system. Two important conclusions have been obtained for the Soret effect in multicomponent silicate melts: first, the SiO_2_ component concentrates in the hot region; and second, heavier isotopes concentrate in the cold region more than lighter isotopes. For the second point, the isotope fractionation can be explained by the classical mechanical collisions between pairs of particles. However, as for the first point, no physical model has been reported to answer why the SiO_2_ component concentrates in the hot region. We try to address this issue by simulating the composition dependence of the Soret effect in CaO–SiO_2_ melts with nonequilibrium molecular dynamics and determining through a comparison of the results with those calculated from the Kempers model that partial molar enthalpy is one of the dominant factors in this phenomenon.

## Introduction

The Soret effect was discovered by C. Ludwig^1^ and tested by C. Soret^2^. Although it has been over 150 years, and the Soret effect can be quantitatively explained by the Chapman’s theory^3^ for the case of molecular gases, the mechanism of the Soret effect in liquid remains controversial^4–6^. The Soret coefficient indicates whether the components diffuse toward hot or cold region and provides the separation degree of the components between hot and cold regions. Neglecting convection, the flux under a temperature gradient in a binary system can be written as follows^7^:

1 $${J}_{1,x}=-\,\rho [{D}_{{\rm{M}}}(\frac{\partial {n}_{1}}{\partial x})+{n}_{1}(1-{n}_{1}){D}_{{\rm{T}}}(\frac{\partial T}{\partial x})],$$

where *x* is the position, *n*_1_ is the mole fraction of the species 1, *ρ* is the mass density, *T* is the temperature, and *D*_M_ and *D*_T_ are the mutual and thermal diffusion coefficients, respectively. In the steady state, the flux *J* = 0 and the Soret coefficient can be written as follows:

2 $${\sigma }_{{\rm{soret}},1}=\frac{{D}_{{\rm{T}}}}{{D}_{{\rm{M}}}}=-\,\frac{1}{{n}_{1}(1-{n}_{1})}\frac{\partial {n}_{1}}{\partial T}\,.$$

A positive Soret coefficient means that the diffusion species concentrate in the cold region, whereas a negative value means they concentrate in the hot region. The relation ${\sigma }_{{\rm{soret}},1}=-\,{\sigma }_{{\rm{soret}},2}$ should be valid in the binary system.

The Soret effect in silicate melts is important in the field of glass and earth sciences, where silicate is a representative component, because it causes the spatial inhomogeneity of the composition in a glass melting container and in the Earth’s interior. Many reports on the Soret effect of silicate melts were released^8–12^, but the dominant factor to determine the Soret coefficients remained controversial. In 2010, Huang *et al*. suggested that the Soret coefficient in silicate melts is expressed by an additive function of the mass- and chemical-effect terms. The mass-effect term is expressed by the variable of mass, charge, and radius of ion. However, the explicit function for the chemical-effect term is unclear^10^. In 2011, Dominiques *et al*. suggested that the electric energy barrier and the vibrational zero-point energy in the activation process of ion diffusion are important factors in determining the Soret coefficient^11^. When tackling this issue, determining the diffusion species in silicate melts is difficult because silicate melts generally contain many structure types (e.g., bridging, nonbridging, and free oxygen, Q_n_ unit (n is the number of bridging oxygen per SiO_4_ unit), and network ring size^13^). Furthermore, the chemical reaction of the Si–O network^14^ and the electric neutrality constraint among ions^15^ make the diffusion process complicated. The diffusion and viscous flow units in silicate melts are also unclear. Therefore, it is difficult to employ the kinetic approach, as similar was done for the Artola^4^ and Eslamian^5^ models, where the Soret coefficient was discussed using the activation energy of the diffusion or viscous flow process of molecular liquid. There has been an approach from a different angle; Kempers model^16,17^ is a thermodynamic model mainly used for the molecular gas and liquid system. This model will be effective in dealing with the problem of silicate melt complexity because it does not need to determine the diffusion species. Kempers said that factors contributing the Soret effect are a function of thermodynamic parameters, such as the partial molar enthalpy, partial molar volume, and chemical potential.

The present study discusses the dominant factor contributing to the Soret effect in a CaO–SiO_2_ system. We use the nonequilibrium molecular dynamics (NEMD) simulation since we can neglect the convection effect caused by gravity and surface tension. Inspired by Kempers^16,17^, we also employ a thermodynamic approach to the Soret effect in silicate melts. We then compare the simulation result with that of a Kempers model, where we take the segregation limit as the standard state of the thermodynamic parameters to adjust the original Kempers model^16,17^ to the silicate melts.

## Results

### Soret coefficient calculated from the NEMD simulation

We simulated the Soret effect of mCaO-(1 − m)SiO_2_ (m = 0.5, 0.6, 0.7, 0.8, and 0.9) melts with the NEMD simulation using approximately 12000 particles at pressure of about 100 MPa(see Method section). The hot region and cold region in the simulation box was kept to be 2200 K and 1800 K, respectively, and linear temperature gradient was obtained. We conducted 22 simulations with different compositions and initial ion positions as shown in Table 1. First, we focus on Run No. 1~21. We waited for the time period of *θ* under temperature gradient before sampling the mole-fraction distribution, where *θ* is characteristic time described in Methods section. The sampling period was 2*θ*.

**Table 1:** Summary of the NEMD conditions and results. The values in brackets in the average row are the standard deviation.

Run No.	composition	Initial ion positions	Ion number in a simulation box				Pressure / MPa	Simulation period		σ_SiO2_ /10^−4^ K^−1^
			Si	Ca	O	Total		Waiting period under temperature gradient before sampling / ns	Sampling period / ns	
1	0.9CaO-0.1SiO_2_	1	600	5400	6600	12600	148.5	2.38	4.75	3.83
2	0.9CaO-0.1SiO_2_	2	600	5400	6600	12600	146.1	2.38	4.75	1.34
3	0.9CaO-0.1SiO_2_	3	600	5400	6600	12600	144.3	2.38	4.75	1.47
4	0.9CaO-0.1SiO_2_	4	600	5400	6600	12600	146.0	2.38	4.75	3.96
5	0.9CaO-0.1SiO_2_	5	600	5400	6600	12600	144.5	2.38	4.75	1.29
6	0.9CaO-0.1SiO_2_	6	600	5400	6600	12600	146.1	2.38	4.75	2.16
7	0.9CaO-0.1SiO_2_	7	600	5400	6600	12600	147.6	2.38	4.75	2.70
Average No.1~7							146.2 (1.5)			2.40 (1.14)
8	0.8CaO-0.2SiO_2_	8	1120	4480	6720	12320	130.6	2.76	5.52	0.23
9	0.8CaO-0.2SiO_2_	9	1120	4480	6720	12320	130.1	2.76	5.52	0.48
10	0.8CaO-0.2SiO_2_	10	1120	4480	6720	12320	133.0	2.76	5.52	1.82
11	0.8CaO-0.2SiO_2_	11	1120	4480	6720	12320	130.7	2.76	5.52	1.10
12	0.8CaO-0.2SiO_2_	12	1120	4480	6720	12320	128.5	2.76	5.52	−0.99
Average No.8~12							130.6 (1.6)			0.53(1.04)
13	0.7CaO-0.3SiO_2_	13	1620	3780	7020	12420	138.2	4.04	8.08	−2.97
14	0.7CaO-0.3SiO_2_	14	1620	3780	7020	12420	138.0	4.04	8.08	−1.70
15	0.7CaO-0.3SiO_2_	15	1620	3780	7020	12420	139.4	4.04	8.08	−1.93
Average No.13~15							138.6 (0.8)			−2.20(0.67)
16	0.6CaO-0.4SiO_2_	16	2080	3120	7280	12480	139.2	6.45	12.90	−3.79
17	0.6CaO-0.4SiO_2_	17	2080	3120	7280	12480	138.9	6.45	12.90	−4.23
18	0.6CaO-0.4SiO_2_	18	2080	3120	7280	12480	139.6	6.45	12.90	−3.43
Average No.16~18							139.2 (0.4)			−3.82(0.40)
19	0.5CaO-0.5SiO_2_	19	2500	2500	7500	12500	132.0	8.79	17.58	−7.71
20	0.5CaO-0.5SiO_2_	20	2500	2500	7500	12500	130.4	8.79	17.58	−8.70
21	0.5CaO-0.5SiO_2_	21	2500	2500	7500	12500	131.9	8.79	17.58	−8.28
Average No.19~21							131.4 (0.9)			−8.29(0.52)
22^*1^	0.9CaO-0.1SiO_2_	22	600	5400	6600	12600	143.6	2.38	4.75	0.96
							145.6		14.25	1.47
							142.0		23.75	1.93
							143.1		33.25	2.30
							144.3		42.75	1.90

^*1^Different sampling time in a run.

**Figure 1:**
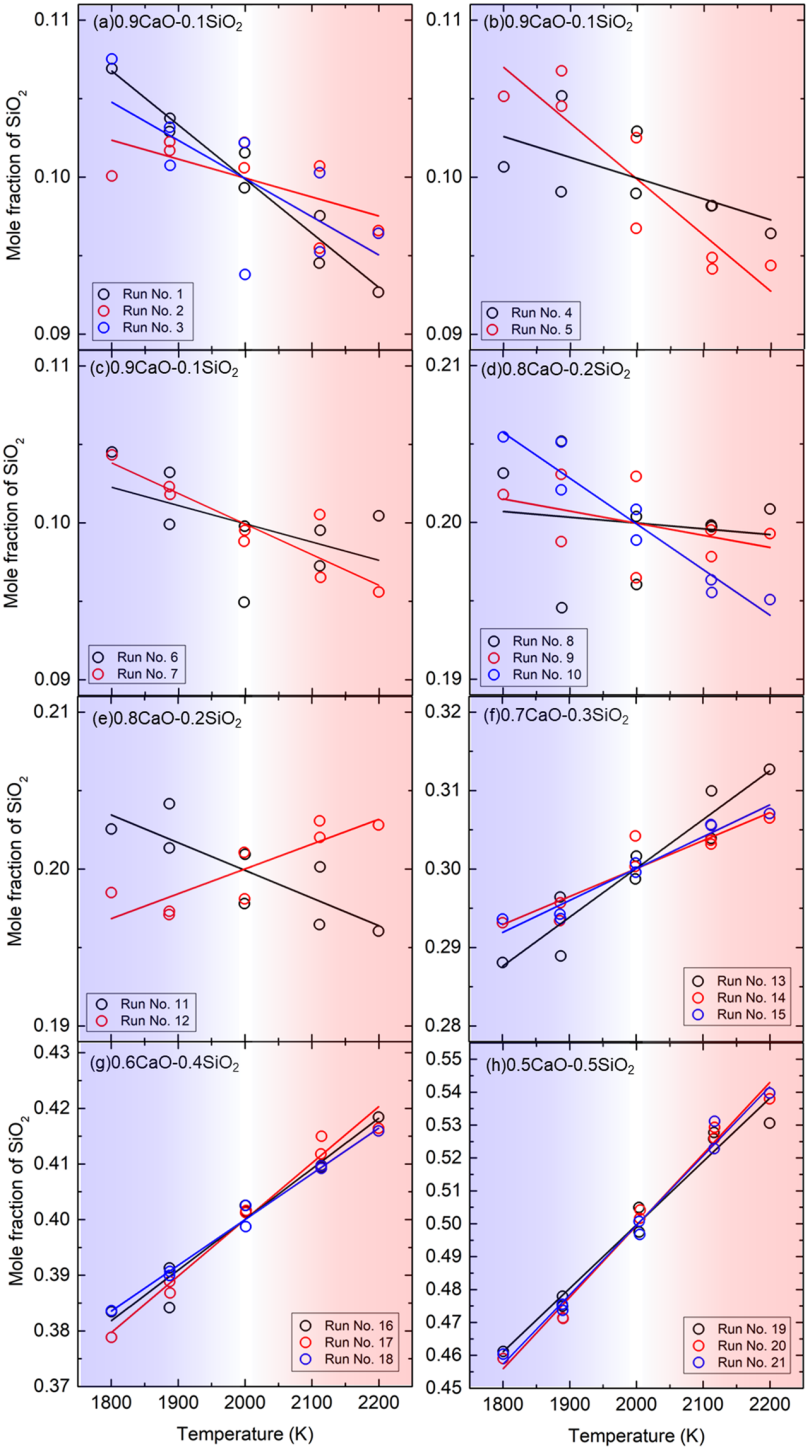
Mole-fraction distributions and fitted lines of SiO_2_ obtained with NEMD. (**a–c**)0.9CaO-0.1SiO_2_. (**d–e**)0.8CaO-0.2SiO_2_. (**f**)0.7CaO-0.3SiO_2_. (**g**)0.8CaO-0.2SiO_2_. (**h**) 0.9CaO-0.1SiO_2_. The 21 simulations were conducted for CaO-SiO_2_ system, and initial ion positions are different from each other. The waiting and sampling period are *θ* and 2*θ*, respectively.

**Figure 2:**
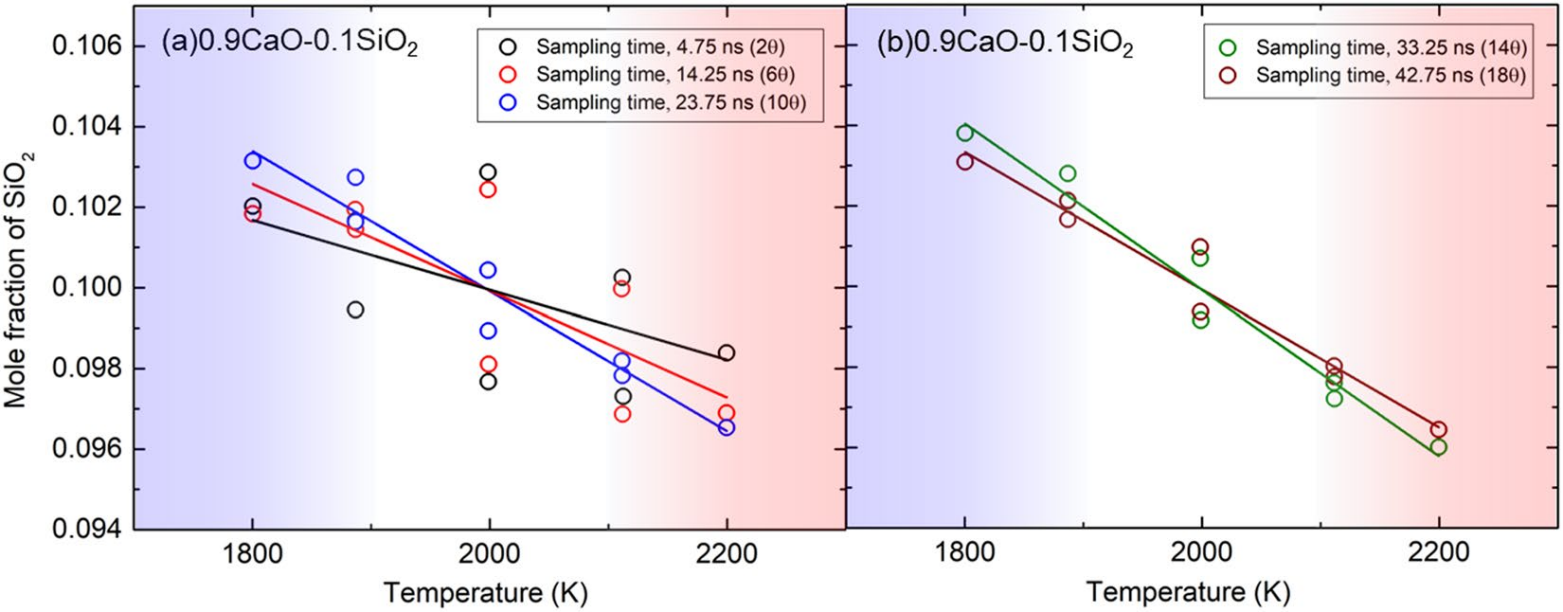
NEMD results obtained with different sampling times for 0.9CaO-0.1SiO_2_. (**a**) sampling time of 2*θ*, 6*θ*, and 10*θ*. (**b**) sampling time of 14*θ* and 18*θ*. This corresponds to the Run No. 22 in Table 1.

Figure 1 shows mole-fraction distribution and fitted line under the temperature gradient. The variation of the mole-fraction distribution even in the same composition of the system is mainly due to the small number of particles in the system (apporoximately 12000 particles). Every fitted line for 0.9CaO-0.1SiO_2_ has a negative gradient, while every fitted line for 0.7CaO-0.3SiO_2_, 0.6CaO-0.4SiO_2_, and 0.7CaO-0.3SiO_2_ has a positive gradient. This means that the SiO_2_-concentrated region is changed with SiO_2_ content of the system; the SiO_2_ concentrates in hot side in a SiO_2_-rich melt, while SiO_2_ concentrates in cold side in a SiO_2_-poor melt. As shown in Fig. 1, the deviation of mole fraction from linear relationship against temperature increases with decreasing SiO_2_ content, which should be due to the small number of Si ions in the simulation box in the SiO_2_-poor melt. To improve statistics for 0.9CaO-0.1SiO_2_, we conducted a simulation with longer sampling times as shown in Fig. 2. The mole-fraction distribution becomes linear with increasing the sampling time. The Si ions will move around the simulation box during the long-time sampling, which will be the reason why we obtained linear relationship. The small difference of plotted data between the 33.25 ns and 42.75 ns in Fig. 2(b) indicates that the concentration distribution is almost converged. This supports the negative gradient of mole fraction of SiO_2_ in the 0.9CaO-0.1SiO_2_ system.

In this study, we take the SiO_2_ and CaO as component 1 and 2, respectively. Since the gradient of the fitted line corresponds to the ∂*n*/∂*T*, by using the Eq. (2), we calculated the Soret coefficients of SiO_2_ component and summarized them in Table 1. The positive and negative values of the Soret coefficient mean the SiO_2_ concentrates in cold region and hot region, respectively. We have reported the Soret effect in 50CaO–50SiO_2_ by a laser irradiation experiment^18^, where the SiO_2_ component concentrated in cold side under temperature gradient. This is qualitatively consistent with the result of NEMD simulation shown in Fig. 1(h).

### Derivation of Kempers model and adjustment to the binary silicate melt

**Figure 3:**
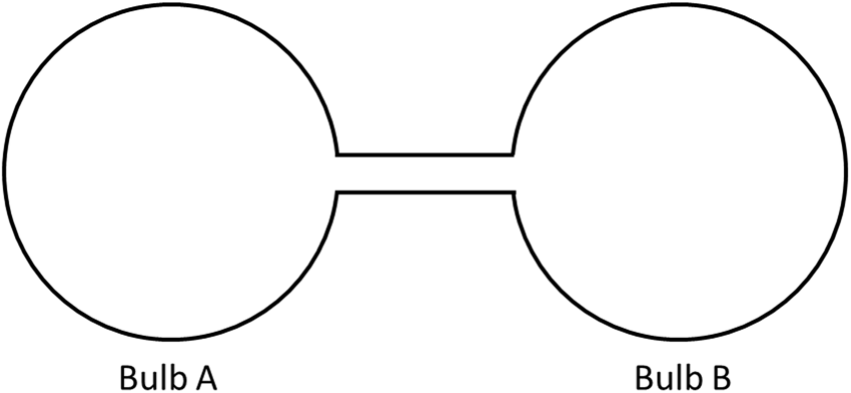
Two-bulb apparatus for the Soret effect.

Kempers proposed a model to calculate the thermodynamic effect of the Soret effect^16,17^. Kempers assumed the two-bulb apparatus, which have equal and constant volumes, as shown in Fig. 3. The system has two components and the bulb A and bulb B are kept to be homogeneous temperature of *T*_A_ = *T* + Δ*T*/2 and *T*_B_ = *T* − Δ*T*/2, respectively. *T* is the average temperature, and Δ*T* is positive value and indicates the temperature difference between bulb A and bulb B.

Kempers assumed that the canonical partition function of the whole system(*Z*_total_) will be maximum at steady state under a temperature gradient:

3 $${\rm{maximum}}\,\{{Z}_{{\rm{total}}}\}.$$

Generally, *Z*_total_ = *z*_A_ × *z*_B_, where *z*_A_ and *z*_B_ are the canonical partition function of the partial system of bulb A and bulb B, respectively. By using the thermodynamic relationship,

4 $$Z=\exp (-\frac{F}{{\rm{R}}T}),$$

where *Z* is canonical partition function, *F* is the Helmholtz free energy, *R* is gas constant, *T* is temperature, we can modify condition (3) to

5 $${\rm{minimum}}\,\{\frac{{F}_{{\rm{A}}}}{{T}_{{\rm{A}}}}+\frac{{F}_{{\rm{B}}}}{{T}_{{\rm{B}}}}\}.$$

Constraint conditions are as follows:

6 $${N}_{{\rm{i}}}^{{\rm{A}}}+{N}_{{\rm{i}}}^{{\rm{B}}}={N}_{{\rm{i}}}^{{\rm{Total}}}\,({\rm{i}}=1,2),$$

7 $${N}_{1}^{{\rm{A}}}{v}_{1}^{{\rm{A}}}+{N}_{2}^{{\rm{A}}}{v}_{2}^{{\rm{A}}}={N}_{1}^{{\rm{B}}}{v}_{1}^{{\rm{B}}}+{N}_{2}^{{\rm{B}}}{v}_{2}^{{\rm{B}}},$$

where *N* is number of the component, *v* is the partial molar volume, i is the number of component. The (6) and (7) express law of conservation of mass and equal volume of each bulb, respectively. By using the method of Lagrange multiplier for (5) under the condition of (6) and (7) and approximation *v*_1_^A^ = *v*_1_^B^ = *v*_1_, *v*_2_^A^ = *v*_2_^B^ = *v*_2_, where *v*_1_ and *v*_2_ are the average partial molar volume of bulb A and bulb B, we can obtain the following condition for steady state under a temperature gradient:

8 $$\frac{\frac{{\mu }_{1}^{{\rm{A}}}}{{T}_{{\rm{A}}}}-\frac{{\mu }_{1}^{{\rm{B}}}}{{T}_{{\rm{B}}}}}{{v}_{1}}=\frac{\frac{{\mu }_{2}^{{\rm{A}}}}{{T}_{{\rm{A}}}}-\frac{{\mu }_{2}^{{\rm{B}}}}{{T}_{{\rm{B}}}}}{{v}_{2}}\,,$$

where *μ* is chemical potential.

By using Taylor expansion, we obtain the following relationship:

9 $$\frac{{\mu }_{i}^{A}}{{T}_{{\rm{A}}}}-\frac{{\mu }_{i}^{B}}{{T}_{{\rm{B}}}}={(\frac{\partial (\frac{{\mu }_{i}}{{\rm{T}}})}{\partial T})}_{p,N,{n}_{1}}{\rm{\Delta }}T+\frac{1}{T}{(\frac{\partial {\mu }_{i}}{\partial {n}_{1}})}_{T,p,N}{\rm{\Delta }}{n}_{1}\,({\rm{i}}=1,2),$$

where *p* is pressure. A relationship for the molar enthalpy of component is:

10 $${h}_{i}=-\,{T}^{2}{(\frac{\partial (\frac{{\mu }_{i}}{{\rm{T}}})}{\partial T})}_{p,N,{n}_{1}}({\rm{i}}=1,2).$$

The delta form of the definition of Eq. (2) is:

11 $${\sigma }_{{\rm{soret}},1}=-\,\frac{1}{{n}_{1}(1-{n}_{1})}\frac{{\rm{\Delta }}{n}_{1}}{{\rm{\Delta }}T}.$$

The Gibbs-Duhem equation in this system is expressed as:

12 $${n}_{1}\frac{\partial {\mu }_{1}}{\partial {n}_{1}}+{n}_{2}\frac{\partial {\mu }_{2}}{\partial {n}_{1}}=0.$$

By the Eqs (9–12), we can obtain the relation between Soret coefficient and thermodynamic parameters:

13 $${\sigma }_{{\rm{soret}},1}=\frac{{v}_{1}{v}_{2}}{{v}_{1}{x}_{1}+{v}_{2}{x}_{2}}\frac{\frac{{h}_{2}}{{v}_{2}}-\frac{{h}_{1}}{{v}_{1}}}{T{n}_{1}\frac{\partial {\mu }_{1}}{\partial {n}_{1}}}.$$

In this study, we assume that the mixing thermodynamic parameters of the two components of binary glass melts should be important for the Soret effect. Under this assumption, we use the departure of thermodynamic parameters from the segregation limit of liquid mixture. We modify the Eq. (13) to the following one:

14 $${\sigma }_{{\rm{soret}},1}=\frac{{v}_{1}{v}_{2}}{{v}_{1}{x}_{1}+{v}_{2}{x}_{2}}\frac{\frac{{h}_{2}-{h}_{2}^{^\circ }}{{v}_{2}}-\frac{{h}_{1}-{h}_{1}^{^\circ }}{{v}_{1}}}{T{n}_{1}\frac{\partial ({\mu }_{1}-{\mu }_{1}^{^\circ })}{\partial {n}_{1}}},$$

where *h*° and *μ*° are partial molar enthalpy and chemical potential of pure liquid state at a given temperature. In this study, we set SiO_2_ and CaO to component 1 and 2, respectively.

### Theoretical value obtained with Kempers model

**Figure 4:**
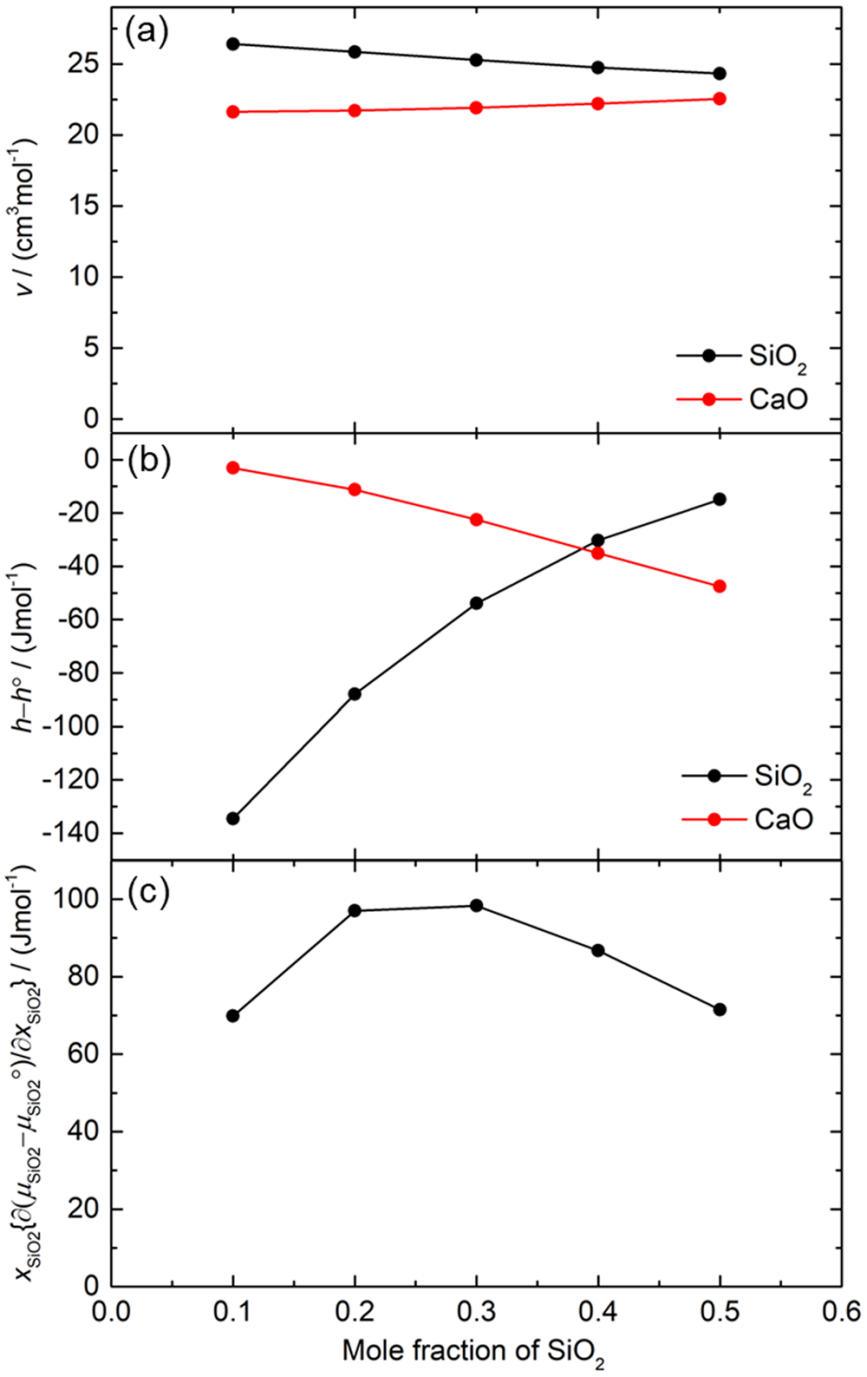
Thermodynamic factors for Kempers model obtained with EMD simulation under non-temperature gradient condition at 2000K and about 100 MPa. (**a**) Partial molar volume, (**b**) departure of partial molar enthalpy of mixture from pure liquid state, and (**c**) *x*_SiO2_{∂(*μ*_SiO2_ − *μ*_SiO2_°)/∂*x*_SiO2_}.

To obtain the thermodynamic parameters which appears in Eq. (14), separately from the NEMD simulation, we calculated thermodynamic parameters with EMD simulation under same pressure and temperature as NEMD simulation. *V*(volume), *U*(internal energy), *H*(enthalpy), *H*^SL^ (enthalpy of segregation limit), Δ*H*^Mix^(enthalpy of mixing), Δ*S*^Mix^(entropy of mixing), and Δ*G*^Mix^ (Gibbs energy of mixing) of the system under *T* = 2000 K and about *P* = 100 MPa were shown in Table 2. Δ*H*^Mix^ was calculated by *H* − *H*^SL^. Δ*S*^Mix^ was calculated using a model proposed by P.L. Lin^19^. The *H*^SL^ can be calculated by simple addition of enthalpy of the pure liquid state: m*H*_CaO_ + (1 − m)*H*_SiO2_, where m, *H*_CaO_, and *H*_SiO2_ are mole fraction of CaO, enthalpy of pure CaO liquid and pure SiO_2_ liquid, respectively. By using *V*, Δ*H*^Mix^, and Δ*G*^Mix^, we calculated *v*(partial molar volume), *h* − *h°*(departure of partial molar enthalpy from pure liquid state), and *μ* − *μ°* (departure of chemical potential from pure liquid state) of the mixture, respectively. Finally, we obtained Soret coefficient of Kempers model(σ_SiO2_^Kempers^) using Eq. (14). Figure 4 shows the mole-fraction dependence of thermodynamic factors which appear in Eq. (14). The *h* − *h°* of the SiO_2_ increases monotonically with increase of SiO_2_ content, whereas that of the CaO decreases. The *h* − *h°* has cross over point of CaO and SiO_2_ around 0.39SiO_2_. The *v* and *x*_SiO2_{∂(*μ*_SiO2_ − *μ*_SiO2_°)/∂*x*_SiO2_} changes gradually with the SiO_2_ mole fraction.

**Table 2:** Summary of thermodynamic parameters for Kempers model and Soret coefficient obtained by the model. All simulations were conducted at 2000 K and about 100 MPa.

Composition	*P*/MPa	*V*/cm^3^ mol^−1^	*U*/kJ mol^−1^	*H*/kJ mol^−1^	*H*^SL^/kJ mol^−1^	Δ*H*^Mix^ = *H*−*H*^SL^/kJ mol^−1^	Δ*S*^Mix^/J mol^−1^	Δ*G*^Mix^ = Δ*H*^Mix^−*T*Δ*S*^Mix^/kJ mol^−1^
SiO_2_	107.9	23.97	−5006.4	−5006.4	−5006.4	0	0	0
0.4CaO-0.6SiO_2_	134.5	23.58	−3693.6	−3693.6	−3666.6	−27.00	6.55	−40.11
0.5CaO-0.5SiO_2_	138.4	23.43	−3362.6	−3362.6	−3331.6	−30.96	6.49	−43.93
0.6CaO-0.4SiO_2_	141.1	23.21	−3029.9	−3029.9	−2996.7	−33.25	5.92	−45.08
0.7CaO-0.3SiO_2_	139.1	22.92	−2693.6	−2693.6	−2661.7	−31.93	5.05	−42.03
0.8CaO-0.2SiO_2_	131.4	22.55	−2353.4	−2353.4	−2326.7	−26.66	4.07	−34.80
0.9CaO-0.1SiO_2_	149.5	22.09	−2007.9	−2007.9	−1991.8	−16.09	2.66	−21.40
CaO	136.0	21.60	−1656.8	−1656.8	−1656.8	0	0	0
**Composition**	***h***_**CaO**_−***h***_**CaO**_**°/J mol**^−**1**^	***h***_**SiO2**_−***h***_**SiO2**_**°/J mol**^−**1**^	***μ***_**SiO2**_−***μ***_**SiO2**_**°/J mol**^−**1**^	***n***_**SiO2**_**{∂(*μ***_**SiO2**_−***μ***_**SiO2**_**°)/∂*n***_**SiO2**_**}/kJ mol**^−**1**^	***v***_**CaO**_ **/cm** ^**3**^ **mol** ^− **1**^	***v***_**SiO2**_ **/cm** ^**3**^ **mol** ^− **1**^	**σ**_**SiO2**_^**Kempers**^ **/10** ^− **4**^ **K** ^**−1**^	
0.5CaO-0.5SiO_2_	−47.55	−14.86	−31.52	71.49	22.54	24.33	−2.453	
0.6CaO-0.4SiO_2_	−35.08	−30.26	−49.24	86.72	22.20	24.75	−0.488	
0.7CaO-0.3SiO_2_	−22.46	−53.91	−76.12	98.31	21.91	25.27	1.362	
0.8CaO-0.2SiO_2_	−11.23	−87.90	−116.33	97.03	21.72	25.85	3.700	
0.9CaO-0.1SiO_2_	−3.10	−134.50	−175.02	69.86	21.62	26.40	9.153	

The superscript of Mix and SL means conditions of mixture and segregation limit, respectively.

### Summary of the NEMD simulation and the Kempers model

**Figure 5:**
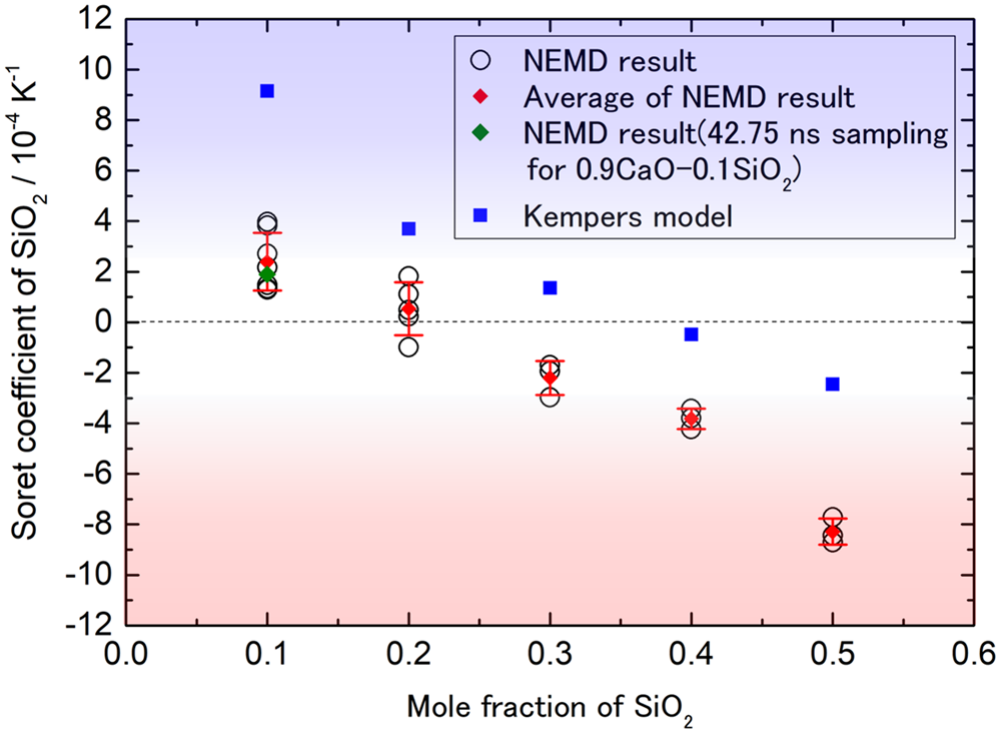
Soret coefficients obtained by NEMD and Kempers model. The error bars indicate standard deviation. The green point indicates the result obtained with the 42.75 ns long-time sampling.

We summarize the results of NEMD and Kempers theoretical model in Fig. 5. Both values monotonically decreases with the increasing mole fraction of SiO_2_ and obtained the turning point of the sign of the Soret coefficient, with the difference of 0.23 for the NEMD and 0.36 for the Kempers model. Both Soret coefficients seem to change in parallel against the mole fraction of SiO_2_. As shown in Fig. 5, the value of lowest edge of the error bar and the value of long-time sampling in 0.9CaO-0.1SiO_2_ are located above zero, which indicates the SiO_2_ concentrates in the cold region. The standard deviation increases with decreasing SiO_2_ content. The large variation of the Soret coefficient in low-SiO_2_ content should be due to the small number of Si ions in the simulation box.

## Discussion

### Comparison of the NEMD result with the Kempers model

The difference between the NEMD simulation and the theoretical model was almost not dependent on the composition, that is, the values changed in parallel. Kempers said that there are two contributions to the Soret effect: thermodynamic contribution from attraction/repulsion and kinetic contribution from collision interaction between components in Soret effect. Based on this idea, the difference between the NEMD simulation and the theoretical model may come from the kinetic factor because we have already considered the thermodynamic contribution in the Kempers model (Eq. (14)). The kinetic factor comes from the collision behavior, and should be expressed as a function of the factors of diffusion species, such as size, mass, and bond strength. Lacks^12^ discussed the contribution of ion mass to the Soret effect caused between isotopes, called isotope fractionation, in silicate melts, and suggested that this contribution to the Soret effect is quantified by scaling relation based on the Chapman–Enskog theory^3^. The qualitative explanation for this is: the penetration depth of the heavier ion from the hot region to the cold region is longer than that of the lighter ion because the former can easily scatter lighter ions. In this study, the diffusion units in the system should be Si−O network, Ca ion, and free O ion. The Si–O network and may act as a heavier diffusion unit than Ca and free O ions, which in turn may contribute to the positive shift to the Soret coefficient of SiO_2_. However, this cannot explain the deviation tendency of the NEMD from the Kempers model. In contrast, analogous to this discussion, the Si–O network behaved as a large-diffusion species compared to Ca and free O ions. The penetration depth of the Si–O network from the hot region to the cold region was shorter than that of the Ca and free O ions because collision frequently occurred in the case of the large-diffusion species. Therefore, the Si–O network will easily concentrate in the hot region through this contribution, and the Soret coefficient of SiO_2_ obtained from the NEMD is shifted to a negative direction from that predicted by the Kempers model. We think that the size effect may be larger than the mass effect in the system.

### Important role of partial molar enthalpy

The sign of the Soret coefficient in Kempers model (Eq. (14)) is determined by the term of (*h*_2_ − *h*_2_°)/*v*_2_− (*h*_1_ − *h*_1_°)/*v*_1_. As shown in Fig. 3, the *h* − *h*° is drastically changed with composition and cross over point is observed around 0.39SiO_2_ and the change of the value of partial molar volume(*v*) is small. (*h*_2_ − *h*_2_°) − (*h*_1_ − *h*_1_°) > 0 in the SiO_2_ content less than 0.39SiO_2_ mole fraction, which indicates that (*h*_2_ − *h*_2_°)/*v*_2_− (*h*_1_ − *h*_1_°)/*v*_1_ > 0 at low SiO_2_ content because the difference between *v*_1_ and *v*_2_ is small in this system. (*h*_2_ − *h*_2_°)/*v*_2_− (*h*_1_ − *h*_1_°)/*v*_1_ > 0 results in positive Soret coefficient of SiO_2_ in the Kempers model, while, in high SiO_2_ content system, (*h*_2_ − *h*_2_°)/*v*_2_− (*h*_1_ − *h*_1_°)/*v*_1_ < 0, which results in negative Soret coefficient. Neglecting the factor contributing to the parallel shift of Soret coefficient and focusing on Eq. (14), we obtain the conclusion that the sign change mainly comes from the magnitude relationship of *h* − *h°* between the two components. In other words, the main factor to determine the diffusion direction of SiO_2_ component is departure of partial molar enthalpy of mixture from pure liquid state. This discussion may be applied to other binary silicate system.

In conclusion, both of NEMD and Kempers model showed a monotonic decrease of the Soret coefficient of SiO_2_ and a sign change of that at low SiO_2_ content. The difference between the two results may be caused by a kinetic factor. According to the Eq. (14) of the Kempers model, mole-fraction dependence of partial molar enthalpy of SiO_2_ and CaO should be the cause of the sign change. As a future work, we should confirm the sign change in Soret coefficient at low SiO_2_ content binary silicate system, experimentally.

## Methods

### NEMD simulation

The simulation method was similar to that in our previous report^20^, but the details had some differences. We employed the potential proposed by Seo^21^. The potential was developed for CaO-SiO_2_ system and is an extended version of the potential for SiO_2_ proposed by Tsuneyuki^22^ to CaO-SiO_2_ system. The potential function is expressed as:

15 $$V(r)=\frac{1}{4{{\rm{\pi }}{\rm{\varepsilon }}}_{0}}\frac{{z}_{{\rm{i}}}{z}_{{\rm{j}}}{{\rm{e}}}^{2}}{r}+{\rm{f}}({b}_{{\rm{i}}}+{b}_{{\rm{j}}})\exp (\frac{{a}_{{\rm{i}}}+{a}_{{\rm{j}}}-r}{{b}_{{\rm{i}}}+{b}_{{\rm{j}}}})-\frac{{c}_{{\rm{i}}}{c}_{{\rm{j}}}}{{r}^{6}},$$

where z is the effective ionic charge, ε_0_ is the dielectric constant of a vacuum, a, b, and c are the characteristic parameters of each ion, f is the standard force, and r is the distance between the ion pair (i and j). The first, second, and third term express the coulomb interaction, short-range repulsion, and dispersion force, respectively.

We used the Leap-frog Verlet algorithm and Ewald sum to treat the Coulomb interaction and the periodic boundary condition^23^. We separated the cubic simulation box with a periodic boundary condition into eight slices (Supplementary Fig. 1). The first slice was a cold slice kept at 1800 K, while the fifth slice was a hot slice kept at 2200 K. An almost linear temperature gradient was produced, and the particle number was approximately 12,000. The characteristic time for the system to the 95% steady state was calculated as follows^9^:

16 $$\theta =\frac{{|{x}_{{\rm{hot}}}-{x}_{{\rm{cold}}}|}^{2}}{\pi {D}_{{\rm{Si}}}}$$

where *x* denotes the coordinates parallel to the temperature gradient in the simulation box, and *D*_Si_ is the self-diffusion coefficient of Si (Supplementary Table 1). After the temperature gradient control started, we waited for *θ* until the system closed to the steady state. Then, in the Run No. 1 ~ 21, we sampled the mole fraction distribution of each atom for the time period of 2*θ*. In the Run No. 22, we sampled with different time periods as shown in Table 1. The system pressure was controlled at approximately 100 MPa in every simulation. The Soret coefficient was calculated from the mole fraction distribution with the gradient of the fitted line of the data in Fig. 1 and Eq. (2).

The information of structure at 2000 K and approximately 100 MPa is shown in Supplementary Fig. 2.

## Electronic supplementary material

Supplementary information
