# Supplementary material for: Role of partial molar enthalpy of oxides on Soret effect in high-temperature CaO–SiO2 melts
Source: Sci Rep. 2018 Oct 19;8:15489. doi: 10.1038/s41598-018-33882-1 (PMC6195551; doi:10.1038/s41598-018-33882-1)
Supplement: Supplementary file 1 — Supplementary information [file 41598_2018_33882_MOESM1_ESM.docx]

**Supplementary Information for**

**Role of partial molar enthalpy of oxides on Soret effect in high-temperature CaO–SiO_2_ melts**

Masahiro Shimizu^1^, Jun Matsuoka^2^, Hiroshi Kato^1^, Takeyuki Kato^1^, Masayuki Nishi^1^, Heidy Visbal^1^, Kohji Nagashima^1^, Masaaki Sakakura^3^, Yasuhiko Shimotsuma^1^, Hiroki Itasaka^4^, Kazuyuki Hirao^5^, and Kiyotaka Miura^1^

^1^Graduate School of Engineering, Kyoto University, Japan

^2^School of Engineering, University of Shiga Prefecture, Japan

^3^Optoelectronics Research Centre, University of Southampton, UK

^4^National Institute of Advanced Industrial Science and Technology, Japan

^5^C-PIER, Kyoto University, Japan


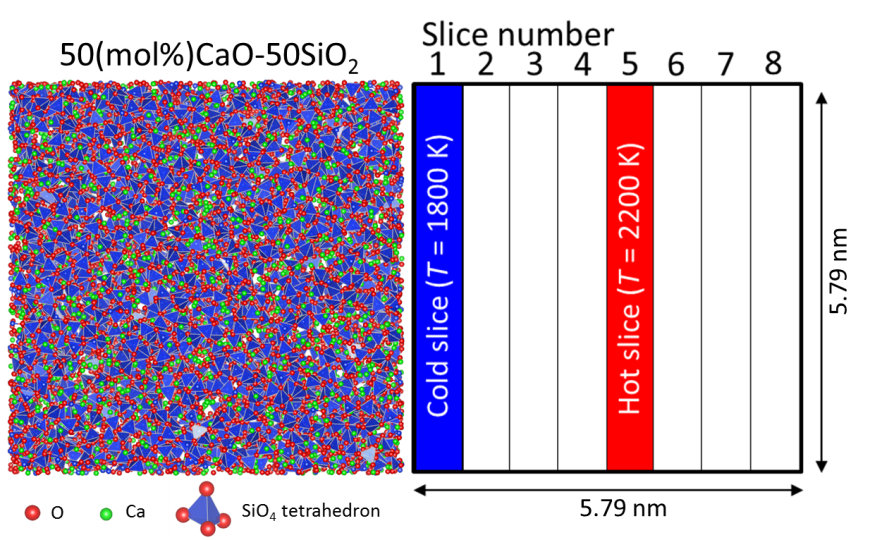


Supplementary Figure 1. An example of the NEMD setup: (left) illustration of the ion configuration in a simulation box (0.5CaO–0.5SiO_2_) and (right) illustration of the temperature control. The first slice is kept at 1800 K, while the fifth slice is kept as a hot slice at 2200 K. The periodic boundary condition is employed.


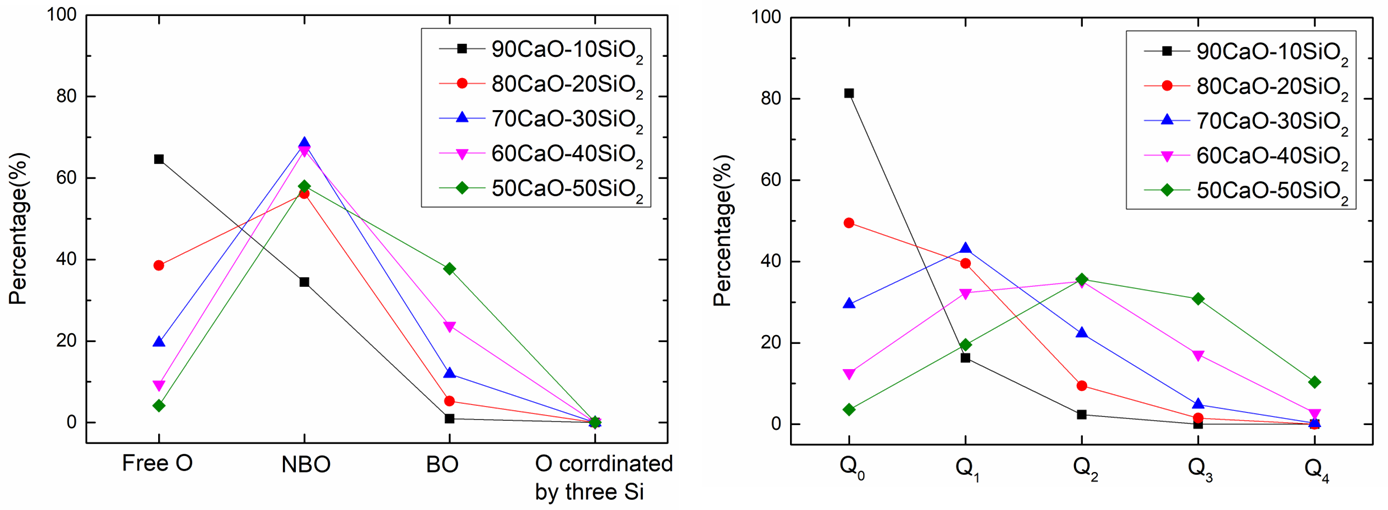


Supplementary Figure 2. (a)Distribution of oxygen species: free, nonbridging, bridging oxygen, oxygen coordinated by three Si. (b)Distribution of Q_n_ unit. All simulations were conducted at 2000 K and approximately 100MPa.

Supplementary Table I. Self-diffusion coefficient of the three ions.

|  |  |  |  |
| --- | --- | --- | --- |
| Composition (mole fraction) | *D*_O_ / cm^2^ s^−1^ | *D*_Si_ / cm^2^ s^−1^ | *D*_Ca_ / cm^2^ s^−1^ |
| 0.5CaO–0.5SiO_2_ | 5.68 × 10^−6^ | 3.04 × 10^−6^ | 1.11 × 10^−5^ |
| 0.6CaO–0.4SiO_2_ | 7.95 × 10^−6^ | 4.23 × 10^−6^ | 1.60 × 10^−5^ |
| 0.7CaO–0.3SiO_2_ | 1.20 × 10^−5^ | 6.87 × 10^−6^ | 2.17 × 10^−5^ |
| 0.8CaO–0.2SiO_2_ | 1.91 × 10^−5^ | 1.02 × 10^−5^ | 2.91 × 10^−5^ |
| 0.9CaO–0.1SiO_2_ | 2.28 × 10^−5^ | 1.22 × 10^−5^ | 3.21 × 10^−5^ |
|  |  |  |  |
